# Views of admitted palliative care patients and their clinicians on corneal donation discussions: a qualitative content analysis of semi-structured interviews

**DOI:** 10.1186/s12904-024-01421-7

**Published:** 2024-04-01

**Authors:** Chirag Patel, Mitchell Nicmanis, Anna Chur-Hansen, Gregory B. Crawford

**Affiliations:** 1 School of Medicine, Faculty of Health and Medical Sciences, University of Adelaide, Adelaide Health, and Medical Sciences Building. Corner of North Terrace and George Street, Adelaide Health Simulation - West End Level 2, Adelaide, 5000 Australia; 2 Northern Adelaide Palliative Service, Northern Adelaide Local Health Network, Adelaide, Australia; 3 School of Psychology, Faculty of Health and Medical Sciences, University of Adelaide, Adelaide, Australia

**Keywords:** Palliative medicine, Corneal donation, Cornea, Eye donation, Qualitative research

## Abstract

**Background:**

There is a severe shortage of corneas for donation, globally, for transplantation and research purposes. One group of individuals who could potentially be donors are those who die within the inpatient palliative care unit. The aim of the study was to understand clinician and patient perceptions of corneal donations and discussion of donation in palliative care units.

**Methods:**

A qualitative design was utilised with data collected through semi-structured interviews and analysed using qualitative content analysis. A total of 46 interviews were undertaken involving inpatient palliative care unit patients (19) and clinicians (27) in three major inpatient palliative care units in South Australia.

**Results:**

Very few patient participants reported being asked about corneal donations during their time in palliative care. Most inpatient palliative care unit clinicians did not raise the topic as they felt other areas of care took precedence. Inpatient palliative care unit patients thought if inpatient palliative care unit clinicians did not raise the topic, then it was not important. There were some differences between patient and clinician views, such as preference about who raises the possibility of donation and when the discussion might occur.

**Conclusions:**

Findings suggest that patients are receptive to discussing corneal donations, but clinicians are not initiating these. This is a missed opportunity for donors and potential recipients. We recommend that clinicians routinely discuss eye donation as part of palliative care.

**Supplementary Information:**

The online version contains supplementary material available at 10.1186/s12904-024-01421-7.

## Introduction

In contrast to other forms of blindness, corneal blindness is curable via corneal transplantation, with overall global mean graft survival rates at 1 and 10 years being 88.6% and 61.2% respectively [1]. There is a severe worldwide shortage of donated corneas for transplantation and research purposes [2–4]. Whilst it is difficult to ascertain how many people are on a waiting list for a transplant, a global survey revealed demand exceeded supply, with only one cornea available for 70 needed [5]. Although malignancy of solid organs is a contraindication for most organ donations, corneas are one of the few tissues that patients with most cancers can donate, haematological malignancies and ocular malignancies being the exceptions [6]. A study in 2020 showed there were barriers to donation from within intensive care but the numbers who went on to donate in intensive care were higher than the numbers from a palliative care unit in 2010 [7, 8].

In 2020 there were 2,277 people in Australia who received a corneal donation from 1318 donors [9]. Approximately two-thirds of these donors were male [10]. Further breakdown of these data show that 57% of donors were between the ages of 60 to 79 years of age while 17.8% were below the age of 50 [10]. Historically this has been the pattern of donation in the 5 years from 2016. Specific data from the 2020 South Australian Eye Bank Service showed a potential of 710 donors from palliative care units but only 32 actual donations (4.5%). This suggests that some states in Australia have higher rates of donation than others. There is a body of literature reporting various sociodemographic factors such as age, gender, education level, and socio-economic status, influence the readiness of a potential donor to consent to organ or tissue donation [11]. There is a need to make more concerted efforts to discuss donations with patients. Australia has an opt-in system for donation, in that a person with decision-making capacity may choose whether to donate their organs and tissue. This can be recorded on the Australian Organ Donor Register. In addition, family members can permit or refuse donation following the death of their relative [12]. In South Australia a dual registration system exists whereby a person can also nominate their wish to donate on their driving license [12].

Achieving a sustained supply of eye tissue is necessary, and given palliative care patients can potentially donate, [13] exploring the perceptions of discussions and donation in these units would provide useful information to potentially increase donation rates. The relative infrequency of corneal donation conversations in palliative care units is known in countries such as the UK, USA, and Australia [3, 8, 14]. Despite efforts to increase discussions and thus donations [15], there is still limited evidence of this occurring [14]. Therefore, palliative care units are a potentially unrealised setting to discuss corneal donation [16]. There is a recent single-site study of patients’ views on corneal donations [17], a study that only involved 9 participants, emphasising their views and timing about how the topic might be discussed. Another limitation of that study was the lack of information regarding the number of participants that were approached, which made it hard to be sure if initial recruitment was truly a census sample as intended [17]. The current study addresses the research question: What are the views of palliative care patients and clinicians about corneal donation discussions? In considering the views of clinicians and patients about corneal donation, the study’s aims and objectives are to understand when to ask, how to ask and in which settings the topic of eye donation might be raised. Patient attitudes to corneal donation have been reported in other countries, but this is, to our knowledge, the first in Australian palliative care patients [3, 18]. The term corneal donation is used throughout this paper whilst acknowledging that in South Australia the whole eye is retrieved from the donor.

## Method

### Design

A qualitative design was constructed with data collected through semi-structured interviews conducted by the first author. The reporting of study methods and outcomes was guided by the Standards for Reporting Qualitative Research (SRQR) [19].

### Setting and participant selection

The study was conducted over 9 months (January 2020–September 2020), with patients and clinicians recruited (doctors, nurses, and social workers) from the only three palliative care units existing within South Australia., In Australia, organisations responsible for organ procurement, overseeing both solid organ and tissue donation processes, depend on notifications from healthcare professionals including doctors, nurses, and social workers regarding potential donations from patients. Given the nature of the palliative care setting, these discussions are largely limited to corneal donations and can occur during different phases of a patient’s illness.

For recruitment of patients, the nursing team leader was asked to provide an information leaflet to those patients admitted to the Palliative Care Unit who met the inclusion criteria (see below), inviting them to participate. If patients were interested, the team leader notified the first author that they had provided consent to be contacted. These patients were approached by the first author and given details of when the interview would take place. They were also given the opportunity to ask further questions. If the nursing team leader deemed the patient who had previously agreed was found to be cognitively impaired or too unwell, they were not further approached. The clinicians were recruited by first contacting the respective heads of unit for the three palliative care sites, who in turn sent out a mass email with the relevant participant information and consent forms, asking to contact the first author directly if they wanted to be interviewed for this study. Once the first author was contacted, a time was organised for the interview to take place. Consent forms were signed by all participants who agreed to be part of the study.

Participants were over the age of 18, English speaking, able to give informed consent (written or verbal), and able to talk for up to 45 min. Social workers and nurses were included in this study as they can initiate discussions around this topic. Clinicians were eligible if they worked with palliative care patients in the in-patient setting. Potential patient participants were not approached if they were not able to converse in English, if they had a delirium, were acutely unwell or actively dying. The research did not aim to assess whether patients would be suitable donors.

### Data collection

All face-to-face interviews were held at the three palliative care units. All patient interviews were conducted face-to-face. Clinician interviews were either face to face or by telephone, depending on preferences and COVID-19 restrictions. An interview guide [20] was used to prompt discussion relevant to the general topic of corneal donation discussions (see Additional file 1). All interviews were conducted by the first author, a medical doctor who had previously worked as a palliative medicine registrar. He had no current clinical role at the recruitment sites. All interviews were recorded electronically and transcribed by the first author. Participants’ rights and privacy were protected by anonymising transcribed data and storing audio recordings on a password protected University server.

### Data analysis

Conventional qualitative content analysis [21] was utilised to inductively organise data into codes that were subsumed by subcategories and overarching categories. Analysis was guided by the research question “What are the views of palliative care patients and clinicians about corneal donation discussions?” The number of interviews was considered sufficient as no new analysis units were identified; this indicated that data saturation was achieved [22]. The analysis was conducted using the software NVivo 12 Plus by the second author, and checked against the raw data by the first, third and fourth authors, for consensus of codes and categories.

### Ethical approval and considerations

The study received approval from the Human Research Ethics Committee at the Central Adelaide Local Health Network (approval number: 12054) which was applicable to all three sites and adhered to the tenets of the Declaration of Helsinki. Specific permission from each site was sought (reference numbers AU/12/5C9A35 and 19–140). During the interviews patients who requested information about becoming a corneal donor were provided with an information pamphlet from the South Australian Eye Bank Service and the Palliative Care Unit was notified of their expressed interest.

## Results

A total of 46 interviews were undertaken involving inpatient palliative care unit patients (*n* = 19) and clinicians (*n* = 27) in three major inpatient palliative care units in South Australia. Of 37 patients approached to participate in the study, 19 consented to be interviewed. Thus 18 patients either declined to participate (*n* = 7) or the interviewer assessed them as not meeting the inclusion criteria due to a change in circumstances (*n* = 11). Patient demographic information is presented in Table 1. Sixty-eight clinicians were approached either by email or directly in person. A total of 27 clinicians responded and consented to be interviewed. Six of the 27 clinician interviews were conducted by telephone. Clinician demographic information is presented in Table 2.

**Table 1:** Demographics for the 19 participating patients

Characteristics	Value
Sex, *n* (%)	
Male	9 (47.4)
Female	10 (52.6)
Unspecified	0 (0.0)
Age (Years)	
Median (SD)	70 (11.4)
Range	39–84
Life-Limiting Illness, *n* (%)	
Breast Cancer	2 (10.5)
Pancreatic Cancer	2 (10.5)
Metastatic Squamous Cell Cancer	2 (10.5)
Prostate Cancer	2 (10.5)
Cervical Cancer	2 (10.5)
Bladder Cancer	1 (5.3)
Endometrial Cancer	1 (5.3)
Lung Cancer	1 (5.3)
Leukaemia	1 (5.3)
Myeloproliferative Disorder	1 (5.3)
Renal Cell Cancer	1 (5.3)
Colorectal Cancer	1 (5.3)
Ovarian Cancer	1 (5.3)
Chronic Obstructive Pulmonary Disease	1 (5.3)

**Table 2:** Demographics for the 27 participating clinicians

Characteristics	Value
Sex, *n* (%)	
Male	9 (33.3)
Female	18 (66.7)
Unspecified	0 (0.0)
Age (Years)	
Median (SD)	42 (10.2)
Range	25–61
Clinical Experience (Years)	
Mean (SD)	6.9 (5.0)
Range	1–18
Profession, *n* (%)	
Nurse	12 (63.2)
Doctor	10 (52.6)
Social worker	5 (26.3)

**Fig. 1:**
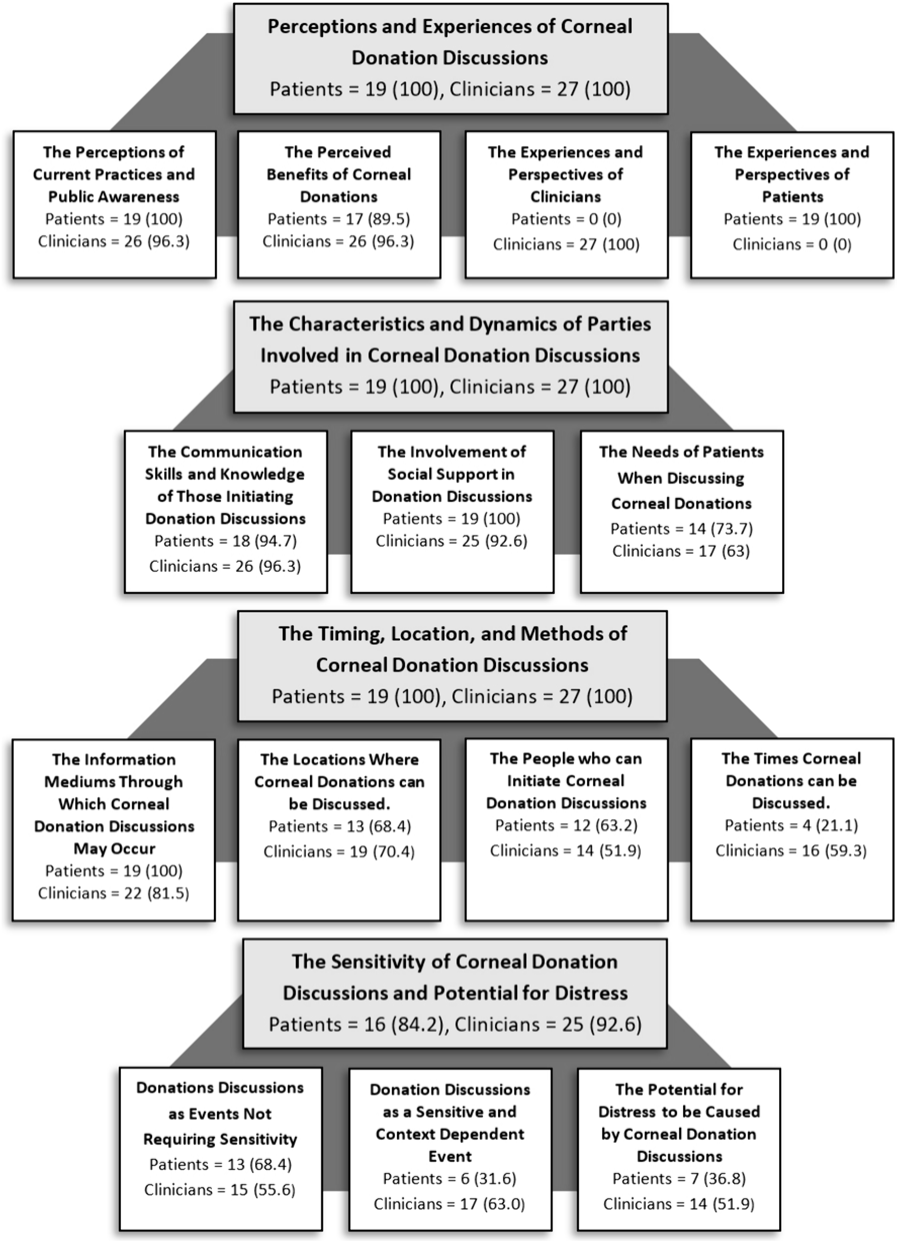
Overarching categories (Light grey) and subcategories occurrence frequencies are represented as *n* (%) and based on the total number of patients (*n* = 19) and clinicians (*n* = 27) that produced corresponding data for each category and subcategory

Data were classified into 97 codes, 14 subcategories, and four overarching categories (see Additional file 2 for the full analysis structure) that described the differing perceptions of palliative care patients and clinicians regarding corneal donation discussions. Additional file 3 provides representative extracts that support the subcategories from the perspective of each participant group. Figure 1 illustrates the four overarching categories and respective subcategories.

### Category one: Understandings and experiences of corneal donation discussions

The first category captured subcategories related to differing and shared understandings and experiences of corneal donations discussions between patients and clinicians. The first subcategory described shared perceptions of patients and clinicians regarding current practices and public awareness. Both groups viewed donation discussions as important. However, they perceived limitations regarding current practices, knowledge, education, and awareness about this topic. Consequently, donations were often not seen as being thought about or discussed:

“I don’t do it all the time as we have so many other things to discuss and to be honest in the centres, we are working in at the moment, it is not embedded in our everyday practice” (Doctor 4).

The second subcategory captured the perceptions shared by both patients and clinicians of the beneficial nature of corneal donations. Both patients and clinicians acknowledged corneal donations as beneficial, providing psychological benefits, a sense of purpose, and legacy for patients with illnesses that limit organ donation options, while offering sight and research contributions to others:

“The fact that I can potentially give sight to someone else is fulfilling for me, even though I know I won’t be around to see it afterwards” (Patient 7).

The third subcategory captured the experiences and perspectives specific to clinicians. While most clinicians stated that they would be comfortable asking about corneal donations, and only four indicated that they would find it difficult, they did not generally ask patients. Instead, they waited for patients to inquire and only discussed donations when there was an opportunity. Clinicians may not ask due to fear and reluctance, or because it was unclear who should initiate these discussions due to possible concerns over professional limitations:

“No as I don’t think it [asking patients about their corneal donation preference] is in the remit of being a nurse … I feel it is outside of my scope” (Nurse 12).

The fourth subcategory captured the experiences and perspectives that were specific to patients. The majority had never been asked about corneal donations, even though they indicated they would like to be asked. Only two patients were unsure about being asked and one indicated that they would not want to donate their organs for research. Patients stated that they were left to find out about corneal donations themselves, and because of a lack of information there was concern that their medical conditions would prevent donation:

“I decided a long time ago even before my cancer that I would be a donor, but now that I have cancer I don’t know if I can, so bringing this up would help me determine this” (Patient 17).

### Category two: The characteristics and dynamics of parties involved in corneal donation discussions

The second category captured descriptions of the desired characteristics of parties involved in donation discussions, along with the interpersonal dynamics that influence these discussions. The first subcategory captured the communication skills and knowledge required to initiate discussions with patients. Discussions required communication skills, medical knowledge, rapport with the patient, knowledge of the patient, gentle character, empathy, fluency in sensitive conversations, and patience, with accounts of these characteristics often overlapping:

“They have to communicate effectively, be empathetic and listen to what I have to say rather than push their views.” (Patient 15).

The second subcategory captured the involvement of social support in donation discussions. It was recommended that either family, friends, or a substitute decision maker was present. While family members could impact the decisions of patients, and there was concern about this, it was indicated by patients that their decision was their own:

“No, my views are my views, so while my family can have their say I know what I want and don’t want.” (Patient 13).

The last subcategory captured the needs of patients during donation discussions. Participants explained that patients should be comfortable with the physical place they are in, the person they are discussing donations with, and their ability to control the conversation. They must also be cognitively able to make decisions and be accepting of their current clinical condition, otherwise the interaction may cause distress:

“There may be some patients who have lived with their disease for a long time and have accepted the outcome, these patients may be ok to discuss [donations] with. However, we also have lots of patients who are newly diagnosed and asking about this you can put a lot of pressure on them and cause distress.” (Nurse 10).

### Category three: The timing, location and method of corneal donation discussions

The third category captured descriptions of the specifically mentioned preferred mediums, locations, people, and times to discuss corneal donations. The first subcategory captured the mediums through which donations could be discussed. Face-to-face was preferred, however, a combination of mediums including written information, telehealth options, videos, and models was desired:

“This will depend on a number of factors for me but if a topic like this was going to be discussed it may be worth giving a warning about it first so you could give some written information and then bring it up on a face-to-face visit” (Patient 7).

The second subcategory captured the different locations where corneal donations could be discussed. Palliative care units, and the homes of patients, were the most frequently mentioned locations. There was little agreement over where the best location was and participants further reported that discussions could occur in the community, outpatient settings, in general practice settings, and in private places:

“I think the place is not strict, I think as long as it is in a private area which is not heard by other people that will be the most important thing” (Nurse 10).

The third subcategory captured who should initiate corneal donation discussions. It was most frequently indicated that doctors should discuss corneal donations with patients due to their knowledge of the procedure. This was followed by nurses, organ donation groups, general practitioners, social workers, chaplains, and medical officers. Clinicians often described multiple professionals collaboratively utilising their specific skills to provide the best outcomes for patients:

“Nurses have the best ongoing relationship [with patients] so they would be ideal, with doctors or people from eye bank then coming in to answer any specific questions” (Doctor 5).

The fourth subcategory captured the times participants indicated were best to discuss corneal donations. Discussions were seen as best early in care, integrated as part of procedural processes either during the preparation of care plans or hospice admission:

“Given the fact there have been no conversations with me, a trigger would be needed so maybe when I had my ACD [advance care directive] discussion, that would have been an ideal opportunity, or even when I am discussing the goals of care when I come into hospital” (Patient 11).

### Category four: The sensitivity of corneal donation discussions and potential for distress

While the third category detailed specific preferences for mediums, locations, people, and times to discuss corneal donations, the final category encompassed contrasting views on the approach to these discussions and the possibility of them causing distress. The first subcategory captured descriptions that depicted corneal donation discussions as events where any extra sensitivity is not required by those initiating the discussion. Participants indicated that discussions could be initiated by anyone, could occur anywhere, and at any time. Discussions could be seen as informal events, not requiring sensitive planning, and were no different from other clinical decisions:

“In my cancer journey I have had to make some difficult decisions around my care. I don’t see this as any more difficult than other issues I have faced. I am quite comfortable making these decisions” (Patient 9).

On the contrary, the second subcategory captured descriptions that indicated these discussions were sensitive and highly context dependent, requiring careful planning and timing. For participants who identified the discussion as sensitive, the specifics about planning these discussions were unclear. Participants referred to a nondescript “right time” and “right person” for these discussions:

“I would want the right time and person to discuss this with me, those would be my only issues” (Patient 8).

The third subcategory captured the potential for these events to cause distress for patients. While four clinicians viewed these discussions as posing no risk to patients, participants indicated that this was a potentially difficult topic to discuss. Discussing donations could be particularly distressing for younger patients and may cause patients to realise their own mortality:

“If I was the patient, I may feel a little unhappy as I am here for treatment, but this conversation may imply that I am dying and that am I being asked because you would like me to die quickly so that my cornea can be taken?” (Nurse 11).

## Discussion

This study provides further evidence that patients are open to having discussions about and donation of corneas [14, 17, 18, 23]. However, it is important to note that a lack of knowledge on this process from a patient perspective may influence the decisions they make on donation [24]. Two studies considering clinicians’ views was in line with this study’s findings, which specifically related to not raising the topic and lack of knowledge [14, 25]. This study demonstrates that many patients and clinicians are unaware or have real misconceptions about corneal donation. Placing informative posters in a dedicated space within the palliative care unit may allow visibility to the patient and families which may increase awareness. Clinicians generally chose not to ask patients, a view corroborated and expressed by patients. Even raising the topic of donation is thus unlikely to be instigated in palliative care [18, 20, 25]. If clinicians do not raise it, either due to concerns about eliciting distress, or because it is not seen as their responsibility, patients’ rights for information are lost.

Both patients and clinicians identified favourable times, places, and approaches for these discussions and indicated that they could be started without requiring extra sensitivity. Yet, many participants wanted to have the conversation, “at the right time” with the “right people” What these factors mean, require further investigation. They will likely mean different things to different stakeholders – a consensus statement for clinicians working in palliative care would be valuable in this regard. There was little consensus over location and the medium in which to discuss. Walker et al. [17] and Cochran et al. [26] found that patients preferred face to face discussions. The present study suggests that providing patients with written information may allow them to develop an understanding and acceptance prior to a telephone or face to face discussion. This may limit the perceived distressing impact of this conversation about which some clinicians were concerned.

Clinicians viewed that their education was limited regarding current practices, and patients felt there was limited public awareness about corneal donations. A common opinion from patients was that clinicians had to be adequately knowledgeable on the subject. For clinicians, this was a common barrier to discussion. Many clinicians and patients knew very little about the whole process, as found in other studies [15, 17, 18, 25, 26]. Whilst clinicians knew that a cancer diagnosis was not a contraindication for corneal donation, many patients did not understand that they could donate their cornea despite a diagnosis of cancer [17, 26].

Clinicians are known at times to be gatekeepers to care including in palliative care [25]. This may impact an eligible person from pursuing corneal donation, because they do not want to distress the patient who may already be distressed about their mortality. While there is nothing obstructing nurses or social workers in discussing this topic, there were barriers to them mentioning this topic which meant less engagement. They either felt it was not within their role to discuss this topic or that it would cause distress to the patient. Discussing corneal donations with people who have not accepted their clinical condition may be particularly distressing. A scoping review in 2021 around this topic revealed that discussing this topic may signal impending death which was a view shared by nurses in this study [3]. More clinicians discussed the perceived distressing nature of this topic compared to patients. However, evidence suggests that patients are not opposed to discussing this topic, but timing is a key consideration. [3, 27]. Discussions were seen as best early in care [18], integrated as part of procedural processes either during the preparation of care plans or hospice admission [3]. Other factors may mediate the perceived distressing impact, such as the communication methods used [27].

Patients felt that a doctor or nurse should initiate the conversations, with the majority indicating doctors. Walker et al. 2018 [17]. believed that doctors were not ideal clinicians to initiate discussions, given the length of their relationships with patients are shorter. Some level of rapport must be present. This was quite apparent in many patient views, as other studies have also demonstrated [17, 18]. Additionally, the ability to communicate sensitively and empathetically is essential, a view also expressed by clinicians. Effective communication can reduce the refusal of donation [28]. There is literature to suggest best practices to improve communication on topics like organ donation discussions, such as utilising the Communicating Effectively About Donation method [28]. This is a skills training program designed to improve the quantity and quality of organ donation discussions. This study displays the value that patients impart to clinicians in asking about and understanding organ donation and in particular corneal donation. Patients had a preference that a doctor would be the best person to raise the topic, yet doctors thought otherwise. This emphasises that clinicians need to understand the patient’s preference to improve the patient’s quality of care [29]. Further research in medical and health professional education is needed, to design, deliver and evaluate communication skill training, to facilitate confidence in having these “difficult conversations”.

Most clinicians and patients recommended that family members and substitute decision makers be present during corneal donation discussions. Choosing whether to donate or not is a challenging and very personal conversation. Most of the patients described that they would want support from family and friends for such discussions. While there was concern from some patients that family members may impact patients’ decisions to donate, patients generally believed that their decision was their own. Studies have shown that families generally are receptive to corneal donation discussions and find the concept to be positive [14, 30]. The importance of family being present is significant and can impact donor rates, as family can ultimately refuse donation following the death of their relative even if the donor provided consent to donate [9].

Corneal donations were seen as having many benefits, for both greater society and for the patients themselves. Commonly, the theme of altruism or “doing good” was expressed by patients, and the positive effect donation could have on themselves was evident in discussions. However, not many patients discussed the benefit for research. Altruistic behaviour is not a new phenomenon, and evidence shows that helping others positively affects mental health [31, 32]. Providing patients with an opportunity to help others or framing it this way could be an avenue to pursue. Clinicians also held the same view and felt it provided patients an opportunity of a legacy and possibly being seen as empowering. Studies from other countries have found similar themes [15, 17, 33]. In the United States of America, there is mandatory reporting of impending death to organ procurement organisations, who then pursue donation discussion either with the patient or the family [34]. This process eliminates the reliance on clinicians to initiate discussions. Given there is literature suggesting that clinicians lack knowledge in this area or fear the patients may find the topic distressing [25], this model of practice could potentially be explored in countries like Australia. Alternatively transitioning to an ‘opt-out’ system, which many other countries use, may also increase donor numbers.

### Limitations of this study

This study has several limitations due to the sample and qualitative approach. The study did not collect sociodemographic data, excluded non-English speaking patients, included clinicians with incomparable levels of work experience, and primarily involved older patients. Whilst qualitative research does not claim representativeness, combined these points potentially introduce participation bias. Future studies may address these limitations to assess potential influences on patient views, as well as further triangulation of qualitative data with stakeholders by including patients, clinicians, and family members. As a qualitative interview study, the transcriptions were not independently verified and intercoder reliability was not assessed. Additionally, the interview guide used was semi-structured and targeted. Future research could benefit from addressing these limitations and using more open-ended interviews to enable a broader and deeper sharing of participants' perspectives.

## Conclusion

This study demonstrates that corneal donation discussions are not distressing for all patients. Palliative care doctors are ideally placed to discuss this topic, along with other members of the health professional team, where there is rapport, but adequate education is imperative to give them the confidence and knowledge to initiate discussions. In addition, embedding this important topic early in the medical school curriculum may help bridge this gap. Advance care planning provides an opportunity for this discussion, or a trigger reminder on an admission proforma could be an alternative option. Due to conflicting opinions over whether discussions are routine or highly sensitive events, it was viewed by some that these discussions might be suited to being part of procedural process. For example, creating a notification system from the organ donation panel to highlight when patients are admitted to the palliative care unit could be explored and thus act as a trigger and reminder that a discussion about corneal donation might be considered if it is clinically appropriate and relevant. A consensus statement to guide discussions may be a useful next step.

### Supplementary Information

**Supplementary Material 1.**

**Supplementary Material 2.**

**Supplementary Material 3.**

## Data Availability

We have included the full analysis structure in this submission. We did not gain participant consent for sharing interview transcripts. Parts of these recordings contain confidential information. These audio recordings are stored on password-protected computers at the palliative care units.

## References

[1] Qureshi S, Dohlman TH (2023). Penetrating Keratoplasty: Indications and graft survival by geographic region. Semin Ophthalmol.

[2] Williams AM, Allingham RR, Beckwith HS, Liu PJ, Santiago-Turla C, Muir KW (2013). Patient and family attitudes about an eye donation registry for research. Curr Eye Res.

[3] Madi-Segwagwe BC, Bracher M, Myall M, Long-Sutehall T (2021). Barriers and facilitators to eye donation in hospice and palliative care settings: A scoping review. Palliat Med Rep.

[4] Long-Sutehall T, Madi-Segwagwe BC, Hurlow A, Faull C, Rayment C, Jacob F, Wale J, Short J, Johnston J, Georgiade K et al: The potential for eye donation from hospice and palliative care clinical settings in England: a retrospective case note review of deceased patients' records. Cell Tissue Bank 2022. 10.1007/s10561-022-10036-2 PMC10209221 36322205

[5] Gain P, Jullienne R, He Z, Aldossary M, Acquart S, Cognasse F, Thuret G (2016). Global survey of corneal transplantation and eye banking. JAMA Ophthalmol.

[6] Gillon S, Hurlow A, Rayment C, Zacharias H, Lennard R (2010). Eligibility for corneal donation within the hospice population. Palliat Med.

[7] Cignarella A, Redley B, Bucknall T (2020). Organ donation within the intensive care unit: A retrospective audit. Aust Crit Care.

[8] Roach R, Broadbent AM (2010). Eye donation in Sydney metropolitan palliative care units. J Palliat Med.

[9] Donate Life: Australian donation and transplantation activity report 2020 https://www.donatelife.gov.au/news-events/news/2021/2020-australian-donation-and-transplantation-activity report#:~:text=There%20were%202%2C277%20Australians%20who,national%20program%20began%20in%202009. Accessed 16 November 2023.

[10] Australia and New Zealand Organ Donation Registry: Eye and Tissue Donation https://www.anzdata.org.au/wp-content/uploads/2021/08/s10_tissue_2020_ar_2021_v1.0_20230524.pdf. Accessed 28^th^ February 2024.

[11] Carola V, Morale C, Vincenzo C, Cecchi V, Errico L, Nicolais G (2023). Organ donation: psychosocial factors of the decision-making process. Front Psychol.

[12] Donate Life: All about donation https://www.donatelife.gov.au/all-about-donation. Accessed 25 January 2024.

[13] López-Navidad A, Soler N, Caballero F, Lerma E, Gris O (2007). Corneal transplantations from donors with cancer. Transplantation.

[14] Long-Sutehall T, Zatorska A, Myall M, Faull C, Hurlow A, Mollart S, Rayment C, Short J, Wale J, Winstanley E, Bracher M (2023). Eye donation in hospice and hospital palliative care settings: perceptions, practice, and service development needs – findings from a national survey. BMC Palliat Care.

[15] Weigel S, Stiel S, Schrems-Hösl L, Oetterich K, Ostgathe C, Steigleder T (2017). Hornhautspende in der palliativmedizin – Auswirkung eines standardisierten vorgehens. Zeitschrift für Palliativmedizin.

[16] Williams AM, Muir KW (2018). Awareness and attitudes toward corneal donation: challenges and opportunities. Clin Ophthalmol (Auckland, NZ).

[17] Walker L, Neoh K, Gilkes H, Rayment C (2018). A qualitative study using semi-structured interviews of palliative care patients’ views on corneal donation and the timing of its discussion. Palliat Med.

[18] Edwards P (2005). Corneal donation within palliative care: a review of the literature. Int J Palliat Nurs.

[19] O'Brien BC, Harris IB, Beckman TJ, Reed DA, Cook DA (2014). Standards for reporting qualitative research: a synthesis of recommendations. Acad Med.

[20] Wells J, Sque M (2002). 'Living choice': the commitment to tissue donation in palliative care. Int J Palliat Nurs.

[21] Hsieh HF, Shannon SE (2005). Three approaches to qualitative content analysis. Qual Health Res.

[22] Saunders B, Sim J, Kingstone T, Baker S, Waterfield J, Bartlam B, Burroughs H, Jinks C (2018). Saturation in qualitative research: exploring its conceptualization and operationalization. Qual Quant.

[23] Stiel S, Hermel M, Radbruch L (2011). Cornea donation from patients deceased at a palliative care unit. Palliat Med.

[24] Lawlor M, Kerridge I, Ankeny R, Dobbins TA, Billson F (2010). Specific unwillingness to donate eyes: the impact of disfigurement, knowledge and procurement on corneal donation. Am J Transplant.

[25] Gillon S, Hurlow A, Rayment C, Zacharias H, Lennard R (2012). Obstacles to corneal donation amongst hospice inpatients: A questionnaire survey of multi-disciplinary team member’s attitudes, knowledge, practice and experience. Palliat Med.

[26] Cochran L, Shorthose K: 71 How and when to ask cancer patients with a palliative diagnosis about corneal donation. A questionnaire based study. BMJ Support Palliat Care 2021. 11(1):34-34.

[27] Wale J, Arthur A, Faull C (2014). An analysis of knowledge and attitudes of hospice staff towards organ and tissue donation. BMJ Support Palliat Care.

[28] Siminoff LA, Traino HM, Genderson MW: Communicating Effectively about Organ Donation: A randomized trial of a behavioral communication intervention to improve discussions about donation. Transplant Direct 2015, 1(2). 10.1097/TXD.0000000000000513 PMC4486302 26146659

[29] Say RE, Thomson R (2003). The importance of patient preferences in treatment decisions–challenges for doctors. BMJ.

[30] Carey I, Forbes K (2003). The experiences of donor families in the hospice. Palliat Med.

[31] Schwartz C, Meisenhelder JB, Ma Y, Reed G: Altruistic social interest behaviors are associated with better mental health. Psychosom Med 2003, 65(5). 10.1097/01.psy.0000079378.39062.d4 14508020

[32] Carney EF (2017). The psychology of extraordinary altruism. Nat Rev Nephrol.

[33] Yew Y-W, Saw S-M (2005). Pan JC-H, Shen H-M, Lwin M, Yew M-S, Heng W-J: Knowledge and beliefs on corneal donation in Singapore adults. Br J Ophthalmol.

[34] Glazier AK (2018). Organ donation and the principles of gift law. Clin J Am Soc Nephrol.

