# Supplementary material for: Views of admitted palliative care patients and their clinicians on corneal donation discussions: a qualitative content analysis of semi-structured interviews
Source: BMC Palliat Care. 2024 Apr 1;23:85. doi: 10.1186/s12904-024-01421-7 (PMC10983643; doi:10.1186/s12904-024-01421-7)
Supplement: Supplementary file 1 — Supplementary Material 1. [file 12904_2024_1421_MOESM1_ESM.docx]

**Additional File 1**

**Overall Interview Guide for Patient and Clinician**

- Understanding of corneal donation in palliative care.
- Personal/professional opinions about discussing corneal donations with patients.
- Perceptions of the benefit to patients being informed about corneal donations.
- Views on how, when, where and by whom patients should be informed on the topic of corneal donations.
- Perceptions on any support required during discussions.
- Views on the skills required to have discussions.
- Any concerns about corneal donation or discussions.

**Patient**

- Have you ever been asked about cornea donations by a palliative care health professional?
- What do you know about corneal donation and the process involved?
- Is it important/beneficial to discuss corneal donations in a palliative care setting?
- Do you think that your cancer would affect you donating your corneas?
- Why would patients want to donate their corneas?
- When do you think is a good time to discuss corneal donations?
- How is the best way to discuss corneal donations?
- Where do you think is the best place to discuss corneal donations?
- Who is your preferred person to discuss this topic with?
- What skills do you feel health professionals require to discuss corneal donation?
- Is there anyone you would like to be present at these discussions?
- Would family views affect your views on donating?
- Do you have any concerns about corneal donation discussions?
- Do you have any views on the way forward for corneal donation in palliative care?
- Would you be interested in donating your corneas if you had adequate information available?

**Clinician**

- What do you know about corneal donation and the process involved?
- Is it important/beneficial to discuss corneal donations in a palliative care setting?
- What do you feel about palliative care patients being informed about the option of corneal donation?
- What is currently happening within your clinical areas?
- Do you think that cancer is a contraindication to donating corneas?
- Do you ever discuss corneal donations with your patients? Why or why not?
- Why is it important to donate corneas?
- When do you think is a good time to discuss corneal donations?
- How is the best way to discuss corneal donation?
- Where do you think is the best place to discuss corneal donations?
- Who is your preferred clinician to initiate these discussions?
- What skills do you feel health professionals require to discuss corneal donation?
- Is there anyone you would like to be present at these discussions?
- Do you have any concerns about the corneal donation discussions?
- Would discussing this be difficult for you?
- What do you think are the important issues we have discussed today?
