# Supplementary material for: Views of admitted palliative care patients and their clinicians on corneal donation discussions: a qualitative content analysis of semi-structured interviews
Source: BMC Palliat Care. 2024 Apr 1;23:85. doi: 10.1186/s12904-024-01421-7 (PMC10983643; doi:10.1186/s12904-024-01421-7)
Supplement: Supplementary file 2 — Supplementary Material 2. [file 12904_2024_1421_MOESM2_ESM.docx]

**Additional File 2**

Full Analysis Structure with Categories, Subcategories, and Codes.

| Categories | Subcategories | Codes | *n*_patients_ | *n*_Nurses_ | *n_doctors_* | *n*_Social Workers_ | *N* |
| --- | --- | --- | --- | --- | --- | --- | --- |
| Perceptions and Experiences of Corneal Donation Discussions |  | ­ | 19 | 12 | 10 | 5 | 46 |
|  | The Perceptions of Current Practices and Public Awareness |  | 19 | 12 | 9 | 5 | 45 |
|  |  | Corneal donations are important to discuss | 15 | 10 | 5 | 4 | 34 |
|  |  | Limited or no knowledge of the donation process | 18 | 7 | 4 | 2 | 31 |
|  |  | Corneal donations are not thought about | 6 | 4 | 6 | 0 | 16 |
|  |  | More medical education and information is needed for clinicians | 0 | 7 | 5 | 3 | 15 |
|  |  | Public information and awareness is limited | 4 | 0 | 2 | 1 | 7 |
|  |  | Current practices regarding discussions need improvement | 0 | 3 | 2 | 0 | 5 |
|  |  | The efficacy of existing written information is potentially limited | 0 | 3 | 1 | 0 | 4 |
|  |  | Corneal donation discussions are not part of current practice | 0 | 0 | 2 | 0 | 2 |
|  |  | Corneal donations need public champions | 0 | 0 | 1 | 0 | 1 |
|  | The Perceived Benefits of Corneal Donations |  | 17 | 11 | 10 | 5 | 43 |
|  |  | Corneal donations are a way of helping others | 10 | 7 | 8 | 4 | 29 |
|  |  | Corneal donations are a way of saving the sight of others | 8 | 5 | 2 | 1 | 16 |
|  |  | Knowing you can donate provides psychological benefits | 6 | 4 | 3 | 2 | 15 |
|  |  | Corneal donations are a way of helping research | 2 | 1 | 4 | 1 | 8 |
|  |  | Corneal donations are a way to have a legacy | 1 | 2 | 3 | 2 | 8 |
|  |  | Corneal donations give people who cannot donate other organs an option | 0 | 2 | 3 | 2 | 7 |
|  |  | Corneal donations are a way of contributing to society | 0 | 1 | 2 | 2 | 5 |
|  |  | Corneal donations provide a sense of purpose | 1 | 1 | 1 | 1 | 4 |
|  |  | Corneal donations can be beneficial for the families of patients | 1 | 0 | 2 | 0 | 3 |
|  |  | Corneal donations are a way of honouring donors | 0 | 0 | 1 | 1 | 2 |
|  | The Experiences and Perspectives of Clinicians |  | 0 | 12 | 10 | 5 | 27 |
|  |  | Would be comfortable asking about donation preferences | 0 | 9 | 8 | 2 | 19 |
|  |  | Does not ask patients about donation preferences | 0 | 5 | 3 | 1 | 9 |
|  |  | Clinicians generally do not discuss donations | 0 | 3 | 5 | 0 | 8 |
|  |  | Waits for patients to discuss corneal donations | 0 | 4 | 2 | 2 | 8 |
|  |  | Professional limitations in discussing donations | 0 | 4 | 0 | 2 | 6 |
|  |  | Sometimes asks about donations | 0 | 1 | 1 | 3 | 5 |
|  |  | Would find it difficult to discuss donations | 0 | 3 | 1 | 0 | 4 |
|  |  | Only discusses donations when there is an opportunity | 0 | 1 | 1 | 0 | 2 |
|  |  | Clinicians may be afraid or reluctant to ask patients | 0 | 1 | 0 | 0 | 1 |
|  | The Experiences and Perspectives of Patients |  | 19 | 0 | 0 | 0 | 19 |
|  |  | Has never been asked about corneal donations | 18 | 0 | 0 | 0 | 18 |
|  |  | Would like to be asked about donating | 13 | 0 | 0 | 0 | 13 |
|  |  | Worried that their condition will prevent organ donation | 4 | 0 | 0 | 0 | 4 |
|  |  | Patients are left to find out about donations themselves | 3 | 0 | 0 | 0 | 3 |
|  |  | Unsure about donations or being asked about donating | 2 | 0 | 0 | 0 | 2 |
|  |  | Would not want to donate for research | 1 | 0 | 0 | 0 | 1 |
| The Characteristics and Dynamics of Parties Involved in Corneal Donation Discussions |  |  | 19 | 12 | 10 | 5 | 46 |
|  | The Communication Skills and Knowledge of Those Initiating Donation Discussions |  | 18 | 11 | 10 | 5 | 44 |
|  |  | Good communication skills are needed to initiate discussions | 11 | 10 | 6 | 4 | 31 |
|  |  | Sufficient medical knowledge is needed to initiate discussions | 7 | 7 | 7 | 3 | 24 |
|  |  | Good rapport with patients is need to initiate discussions | 7 | 4 | 4 | 1 | 16 |
|  |  | Sufficient knowledge of the patient is needed to initiate discussions | 7 | 2 | 2 | 0 | 11 |
|  |  | The people initiating discussions should demonstrate gentle character traits | 5 | 4 | 0 | 1 | 10 |
|  |  | Empathy is needed when discussing donations | 4 | 3 | 2 | 0 | 9 |
|  |  | Fluency in sensitive conversations is required to initiate discussions | 1 | 4 | 1 | 2 | 8 |
|  |  | Patience is needed when discussing donations | 1 | 0 | 0 | 0 | 1 |
|  | The Involvement of Social Support in Donation Discussions |  | 19 | 11 | 9 | 5 | 44 |
|  |  | Would like family involved or present during discussions | 15 | 7 | 6 | 3 | 31 |
|  |  | Family preferences would not impact donation decisions | 15 | 0 | 0 | 0 | 15 |
|  |  | The presence of family members is optional | 0 | 3 | 1 | 2 | 6 |
|  |  | The patient’s substitute decision makers should be present | 0 | 1 | 4 | 0 | 5 |
|  |  | Would like friends present during the discussions | 3 | 0 | 1 | 0 | 4 |
|  |  | Concerns about families perceptions of donation | 2 | 0 | 0 | 0 | 2 |
|  |  | Family preferences would impact patients’ donation decisions | 2 | 0 | 0 | 0 | 2 |
|  |  | Concerns about family permissions for donation | 0 | 0 | 0 | 1 | 1 |
|  |  | Not sure if family views would impact their decision | 1 | 0 | 0 | 0 | 1 |
|  |  | Regret from declining a family member’s donation decision | 1 | 0 | 0 | 0 | 1 |
|  | The Needs of Patients When Discussing Corneal Donations |  | 14 | 8 | 5 | 4 | 31 |
|  |  | Patients must be accepting of their clinical condition | 14 | 6 | 3 | 3 | 26 |
|  |  | Patients must have the ability to make decisions | 0 | 4 | 2 | 1 | 7 |
|  |  | Discussions should happen in a place the patient feels comfortable | 1 | 2 | 1 | 0 | 4 |
|  |  | Discussions should occur with someone patients’ feel comfortable with | 4 | 0 | 0 | 0 | 4 |
|  |  | Discussions should be controlled by the patient | 1 | 0 | 0 | 2 | 3 |
| The Timing, Location, and Methods of Corneal Donation Discussions |  |  | 19 | 12 | 10 | 5 | 46 |
|  | The Information Mediums Through Which Corneal Donation Discussions may Occur |  | 19 | 9 | 8 | 5 | 41 |
|  |  | Face-to-face discussions can be used to inform patients | 17 | 9 | 7 | 5 | 38 |
|  |  | Multiple information mediums can be used to inform patients | 11 | 6 | 8 | 4 | 29 |
|  |  | Written information can be used to inform patients | 10 | 7 | 7 | 4 | 28 |
|  |  | Telehealth options can be used to inform patients | 0 | 0 | 3 | 1 | 4 |
|  |  | Video information can be used to inform patients | 1 | 0 | 1 | 0 | 2 |
|  |  | Physical models can be used to inform patients | 0 | 0 | 1 | 0 | 1 |
|  | The Locations Where Corneal Donations can be Discussed |  | 13 | 11 | 4 | 4 | 32 |
|  |  | Discussions can occur in palliative care units | 11 | 9 | 2 | 2 | 24 |
|  |  | Discussions can occur at the homes of patients | 8 | 7 | 2 | 3 | 20 |
|  |  | Discussions can occur in the community | 3 | 3 | 0 | 1 | 7 |
|  |  | Discussions can occur in outpatient settings | 1 | 3 | 1 | 1 | 6 |
|  |  | Discussions should occur in a private place | 1 | 3 | 0 | 0 | 4 |
|  |  | Discussions can occur in general practice | 1 | 2 | 0 | 0 | 3 |
|  | The People who can Initiate Corneal Donation Discussions |  | 12 | 7 | 5 | 2 | 26 |
|  |  | Doctors can discuss corneal donations | 9 | 4 | 2 | 1 | 16 |
|  |  | Nurses can discuss corneal donations | 1 | 3 | 1 | 1 | 6 |
|  |  | Organ donation groups can discuss corneal donations | 0 | 2 | 2 | 0 | 4 |
|  |  | General practitioners can discuss corneal donations | 1 | 1 | 1 | 0 | 3 |
|  |  | Social workers can discuss corneal donations | 1 | 1 | 0 | 0 | 2 |
|  |  | Chaplains can discuss corneal donations | 0 | 0 | 1 | 0 | 1 |
|  |  | Medical officers can discuss corneal donations | 0 | 0 | 0 | 1 | 1 |
|  | The Times Corneal Donations can be Discussed |  | 4 | 5 | 8 | 3 | 20 |
|  |  | Discussions can happen early in care | 2 | 3 | 4 | 1 | 10 |
|  |  | Discussions can be part of preparing patient care plans | 2 | 1 | 5 | 1 | 9 |
|  |  | Discussions can be part of admission processes | 0 | 2 | 5 | 1 | 8 |
| The Sensitivity of Corneal Donation Discussions and Potential for Distress |  |  | 16 | 12 | 9 | 4 | 41 |
|  | Donations Discussions as Events not Requiring Sensitivity |  | 13 | 5 | 6 | 4 | 28 |
|  |  | Discussion can be initiated by anyone | 3 | 5 | 4 | 0 | 12 |
|  |  | Discussions can occur anywhere | 6 | 0 | 4 | 2 | 12 |
|  |  | Discussions can occur at any time | 8 | 0 | 1 | 1 | 10 |
|  |  | Deciding to donate is no different from other decisions | 4 | 0 | 1 | 1 | 6 |
|  |  | Discussions can be informal | 1 | 0 | 0 | 0 | 1 |
|  | Donation Discussions as a Sensitive and Context Dependent Event |  | 6 | 10 | 5 | 2 | 23 |
|  |  | Timing of the discussion is important | 5 | 7 | 3 | 2 | 17 |
|  |  | Discussions should be initiated at the “right time” | 3 | 6 | 1 | 1 | 11 |
|  |  | Best to alert patients before initial discussion | 1 | 3 | 1 | 0 | 5 |
|  |  | Discussions require careful planning | 2 | 1 | 1 | 0 | 4 |
|  |  | Discussions should occur with the “right person” | 2 | 1 | 0 | 0 | 3 |
|  | The Potential for Distress to be Caused by Corneal Donation Discussions |  | 7 | 6 | 6 | 2 | 21 |
|  |  | Discussing donations is a potentially difficult topic | 7 | 4 | 2 | 2 | 15 |
|  |  | Discussing donations does not pose a risk to patients | 0 | 1 | 3 | 0 | 4 |
|  |  | Discussions may cause patients to realise their own mortality | 0 | 4 | 0 | 0 | 4 |
|  |  | Discussions had too early can be confronting | 0 | 2 | 1 | 1 | 4 |
|  |  | Discussions had too late can be confronting | 0 | 1 | 2 | 1 | 4 |
|  |  | Discussions are difficult for young patients | 2 | 0 | 0 | 0 | 2 |

*Note.* Frequencies are based on the total number of participants who produced accounts corresponding to each code. *n*_patients_ = 19; *n*_Nurses_ = 12; *n_doctors_* = 10; *n*_Social Workers_ = 5; Total = 46.
