# Supplementary material for: Views of admitted palliative care patients and their clinicians on corneal donation discussions: a qualitative content analysis of semi-structured interviews
Source: BMC Palliat Care. 2024 Apr 1;23:85. doi: 10.1186/s12904-024-01421-7 (PMC10983643; doi:10.1186/s12904-024-01421-7)
Supplement: Supplementary file 3 — Supplementary Material 3. [file 12904_2024_1421_MOESM3_ESM.docx]

**Additional File 3**

Further Supplementary Extracts from Participant Groups to Support the Reported Findings for Each Subcategory

| **Categories and Subcategories** | **illustrative Extracts** |
| --- | --- |
| **Category One: Understandings and Experiences of Corneal Donation Discussions** |  |
| The Perceptions of Current Practices and Public Awareness | “It might be a difficult conversation to have, but it is so important. I talk with my nurses and doctors about many things and some conversations are hard and emotional. That doesn’t mean we should avoid it and that it’s wrong.” (Patient 9)  “Most definitely [corneal donations are important] and at the moment I feel it is not done enough.” (Nurse 1)  “I think we all have that responsible to do it and it is just a matter of us having the education to be able to bring it up” (Social Worker 4) |
| The Perceived Benefits of Corneal Donations | “They are told the big C word and suddenly the person would feel they are unhealthy, so the opportunity to donate something to another person would give them a lift and positive spin on their situation” (Nurse 9)  “It’s a way of giving back …Patients are altruistic, and they like to contribute to research and contribute to other people” (Doctor 10)  “There are some patients who I have been involved with previously, they talk about donations in general … and so we explain to them that because of their illness they cannot, but one thing they can donate is their corneas” (Social Worker 4) |
| The Experiences and Perspectives of Clinicians | “It’s something that we don’t ask about and we wait for people to bring it up” (Nurse 2)  “Well, I don’t routinely discuss it [corneal donations] and it is bought up by patients … It is a patient driven conversation” (Doctor 6)  “I usually wait for the patient or relative to bring it up. I don’t bring it up with everyone because I’m a bit gutless” (Social Worker 1) |
| The Experiences and Perspectives of Patients | “Even though I have not thought about it I would have liked someone to discuss with me” (Patient 3)  “I feel discussions on this subject are not very well organised and it is sometimes left to the patient to find out themselves” (Patient 2)  “I have thought about trying to find out more about donation and I wondered if there was anything I could donate given my cancer” (Patient 8) |
| **Category Two: The Characteristics and Dynamics of Parties Involved in Corneal Donation Discussions** |  |
| The Communication Skills and Knowledge of Those Initiating Donation Discussions | “They [the person initiating discussion] need to be knowledgeable and confident in sensitive discussions, so communication and being empathetic springs to my mind.” (Nurse 2)  “Understanding the process is important … Good interpersonal skills and the ability to read the person” (Doctor 2)  “It is like anything we discuss with patient – being able to meet the person where they are at, to respect anything they say about what they want to talk about and don’t want to talk about” (Social Worker 3) |
| The Involvement of Social Support in Donation Discussions | “It is up to the patient, but I think having family there would be useful” (Nurse 2)  “It’s a sensitive subject so having family members present may be a good idea” (Doctor 1)  “Of course, family, if they were part of the conversation and the context of the discussions that took place they are more likely to abide by the patient’s decision” (Social Worker 5) |
| The Needs of Patients When Discussing Corneal Donations | “When the patient accepts what is going on and more difficult conversations are had then it may be the time for example during a ACD [advanced care directive] discussion” (Patient 15)  “It is an important discussion and in the palliative care setting not everyone may be ready to discuss this as some people are not ready for their own death” (Doctor 2)  “Depends on the mindset of the patient – whether they are accepting of their palliative condition” (Social Worker 1) |
| **Category Three: The Timing, Location, and Method of Corneal Donation Discussions** |  |
| The Information Mediums Through Which Corneal Donation Discussions May Occur | “Written communication together with face to face is best – the written communication will reinforce face to face discussions” (Nurse 3)  “I am always big on face to face, so I will have to go with this format personally, but I know that written information is also helpful for these conversations so using both may be the best” (Doctor 4)  “If you want to make it personal then you should have some engaging face to face conversation complemented with some written material” (Social Worker 2) |
| The Locations Where Corneal Donations can be Discussed | “I would be ok with anywhere – home, outpatient, or the hospice” (Patient 11)  “It would be really good potentially as part of information packs we give out …It could be part of a home visit or admission to hospice” (Doctor 5)  “I think in someone’s house it gives the patient control, but I think if they were in a palliative care unit then that is an appropriate place as well” (Social Worker 3) |
| The People who can Initiate Corneal Donation Discussions | “I think doctors have the greatest knowledge so I would go with them” (Patient 7)  “There would need to be someone who understands this process so may be a doctor or even the people who organizes this” (Nurse 5)  “I am inclined to think it fits more in the context of medical so a doctor would be my inclination: so an introduction with the doctor and the follow up could be with nurse or social worker” (Social Worker 5) |
| The Times Corneal Donations can be Discussed | “Apart from this I think the earlier the better as part of information gathering – so people are not suspicious about the trigger – i.e. why are you asking me this now?” (Doctor 2)  “I would think that if they have a terminal diagnosis and a prognosis, then this would be a good time to talk about for example when doing a ACD [advanced care directive]” (Nurse 5)  “It could be that after the patient has had those conversations around goals of care with a doctor and then a social worker could come in later to discuss ACD [advanced care directive] and add in donation information then” (Social Worker 1) |
| **Category Four: The Sensitivity of Corneal Donation Discussions and Potential for Distress** |  |
| Donations discussions as events not requiring sensitivity | “I have learnt that in this area lots of tough conversations are had so it is part and parcel really” (Patient 11)  “No, I don’t think there is a preference, and anyone should be able to ask about this” (Doctor 8)  “I don’t think it is such a taboo topic in that it needs to be carefully skirted around, so outpatients, ward, home are good places. For me anywhere is ok” (Doctor 9) |
| Donation Discussions as a Sensitive and Context Dependent Event | “We have to go gentle as the patient and family may already be stressed, we have to find the right time” (Nurse 4)  “The window of opportunity is small, so we have to get the timing right” (Doctor 7)  “I think that sometimes people are overwhelmed with all they have to deal with and working that into the conversation needs to be timed well and needs to be done sensitively” (Social Worker 5).” |
| The Potential for Distress to be Caused by Corneal Donation Discussions | “This conversation is very difficult and sensitive, so we have to be careful not to bring it up with every single person out of the blue, planning and preparation is important” (Patient 19)  “Being so young I would find it difficult to talk about so we have to acknowledge a lot of factors when we bring up a topic like this” (Patient 12)  “It is rather confrontational for some people and for relatives particularly as it brings about a certain sense of reality about the impending death” (Social Worker 5) |
